# Supporting conflict‐sensitive, locally‐led humanitarianism in Sudan: rebalancing donors' approach to risk

**DOI:** 10.1111/disa.70034

**Published:** 2025-12-17

**Authors:** Becky Carter, Hassan‐Alattar Satti

**Affiliations:** ^1^ Institute of Development Studies University of Sussex United Kingdom; ^2^ Independent Researcher Sudan

**Keywords:** conflict, conflict sensitivity, humanitarian, localisation, risk management, Sudan

## Abstract

Locally‐led mutual aid has been a critical lifeline for many people caught up in Sudan's current conflict. Yet, global humanitarian commitments to empower and support local actors are not being met. Furthermore, remote international organisations risk being insensitive to conflict dynamics and undermining local support systems. Drawing on qualitative research conducted between June 2023 and February 2024 on food and cash transfers in Sudan, this paper finds that diverse grassroots organisations have proved their worth in appalling circumstances, while international support for them remains at a small scale. It argues that localisation and conflict sensitivity agendas can be mutually supportive. Long‐term partnership with, and core funding for, carefully selected local actors can deepen contextual understanding and empower those best placed to manage conflict over the longer term. This requires that donors review their approach to risk management and consider the potential harms of continuing to centralise control over interventions, as well as the opportunities lost in doing so.

## 1 INTRODUCTION

Since April 2023, community groups have provided critical humanitarian support to those affected by the conflict in Sudan. These local initiatives have attracted global attention, with headlines including ‘How mutual aid networks are powering Sudan's humanitarian response’ (Nasir, Rhodes, and Kleinfeld, 2023). Some international donors and aid agencies have committed to supporting this mutual aid response. The formal aid system, however, has provided it with limited funding.

This paper is based on a study conducted between June 2023 and February 2024.^1^ We looked at how grassroots initiatives were responding to the impacts of the conflict and how international aid actors were engaging with these community‐level efforts. We summarise our findings here in relation to two key research questions:

1. What can external aid actors learn from locally‐led initiatives in Sudan to improve the conflict sensitivity of their policy and programming?
2. How can official aid link with and support community mechanisms in Sudan in a conflict‐sensitive way?

We hope that the answers may guide those in a position to respond to the gravity of the situation prevailing in Sudan and encourage them to do so more urgently.

This topic intersects three areas of policy and debate in the humanitarian field. The first is the humanitarian localisation reform agenda (World Humanitarian Summit, 2016; Grand Bargain Localisation Workstream, 2019) and the relationship between global and local within the aid system. The second is the conflict sensitivity agenda, which considers how aid interventions can be designed and delivered to respond to and engage positively with conflict (and peace) dynamics (OECD, 2019; IASC, 2020; UNSDG, 2022). The third is donor risk management: donors' understanding of risk and related mitigating strategies are both shaped by and in turn set the parameters for localised and conflict‐sensitive assistance.

This paper contributes to thinking around humanitarian response in conflict by exploring the relationship between these three themes: localisation; conflict sensitivity; and approaches to risk. Our research adds to the evidence base on why it is proving so hard to reform global humanitarianism to be ‘as local as possible, as international as necessary’ (UN, 2016, p. 30), and identifies how official aid can support grassroots assistance in a manner that minimises the risk of doing harm, engages positively with conflict dynamics, and may even support peace. We find that while localising aid funding and decision‐making is not sufficient to ensure that the assistance has a positive interaction with conflict dynamics, a conflict‐sensitive approach to localisation will deepen contextual understanding and reinforce the capacities of those best placed to mitigate the impacts of conflict over the longer term (Robinson, 2021; Haines and Buchanan, 2023).

Our analysis, though, reveals three critical challenges in the Sudanese context that constrain this type of conflict‐sensitive support to locally‐led initiatives:

1. The shortage of funds, which makes it hard to select aid recipients when needs are widespread. This heightens the risk of fuelling social tension, and weakens implementation capacity.
2. The lack of longer‐term investment in national non‐governmental organisation (NGO) capacities and their ‘bridging’ role between the formal aid system and grassroots structures.
3. The programming pressures of large‐scale, fast‐paced, and logistics‐led operations.

Building on reflections by Caravani et al. (2022), we perceive that donors and aid agencies may instinctively seek greater control over their assistance in conflict‐affected situations where risks become increasingly complex and acute. Yet, such centralisation brings risks of its own. Our research underscores the value of thoroughly comprehending the full set of risks to international assistance in conflict crises, including those that arise from the centralising tendency of external actors.

In the next section of the paper, we investigate the context of the conflict in Sudan and set out the conceptual framing for our three major themes: localisation; conflict sensitivity; and risk management. This is followed by two sections that present our methods and results. We then engage in a discussion of our findings before offering some conclusions.

## 2 CONTEXT OF THE CONFLICT IN SUDAN

### 2.1 Origins, impacts, and humanitarian response

The roots of the crisis lie in the nature of governance in the highly unequal country of Sudan, particularly the exclusionary concentration of power and resources among a network of military, private sector, and political actors. This is compounded by the extent of impunity for previous human rights violations, under former President Omar al‐Bashir as well as the subsequent transitional regime (Cartier, Kahan, and Zukin, 2022; Hoffmann and Lanfranchi, 2023; Jaspars and Oette, 2023; REDRESS, 2023).

The 2019 revolution was preceded by emerging grassroots forms of political organisation and civic action over the preceding decade, in which young women and men found their voice (Abbas and Al Karib, 2023; Abbashar, 2023; El‐Gizouli and Thomas, 2023). Across Sudan, neighbourhood groups coalesced into resistance committees, developing from earlier civic action that included mobilising voters in the 2010 elections, protesting against the reduction of subsidies in 2013, and helping those affected by disasters (The Carter Center, 2021; Marovic and Hayder, 2022; Abbashar, 2023). These resistance committees—‘arguably the largest, youngest and most active political agent in the country’ (El‐Gizouli and Thomas, 2023, p. 5)—mobilised against the al‐Bashir government, embodying and motivating revolutionary spirit in a ‘people's movement’. This led to the military coup that deposed the regime in April 2019, and subsequent demands for a fully civilian government (Abbas and Al Karib, 2023; El‐Gizouli and Thomas, 2023).

The promise of democratic reform was dashed, though, as the power‐sharing agreement between the civilian coalition of the Forces of Freedom and Change and the military prioritised protecting geopolitical interests abroad over meeting the social justice demands of the resistance committees. During this period, the security forces and the paramilitary Rapid Support Forces (RSF) committed atrocities and engaged in crackdowns on protestors and resistance committees (REDRESS, 2023).

Such targeting and exclusion of the grassroots movement of the resistance committees has long been in the making (Osman, 2022). Silencing their voice has been a major tactic of both the civilian and military components of the transitional government. The transitional authorities were grappling with the demands of external powers (Sørbø, 2020), which were often at odds with the demands of the people (Kiwuwa, 2020). A good example of silencing the voice of the grassroots movement pertains to the delays in forming the legislative council that was supposed to be led by the resistance committees. The targeting of grassroot actors, many of which reorganised themselves as emergency response rooms (ERRs), has continued during the war between the Sudanese Armed Forces (SAF) and the RSF, given their legacy of hostilities towards grassroot groups.

Violent conflict erupted between the SAF and the RSF in April 2023. At the time of writing, the fighting is into its third year, with extensive, severe impacts. This paper is based on research undertaken between June 2023 and February 2024. We present here key aspects of the conflict's impact during that period, to set the context that shaped our findings and analysis—for more detail, see Carter and Satti (2025).

The scale of civilian death and injury, and the weaponisation of rape and sexual violence, points to a disregard for international humanitarian law (Amnesty International, 2023), with the rapid escalation and ruthlessness of violence in the region of Darfur (western Sudan) being particularly shocking (Sudan Conflict Observatory, 2023; ACLED, 2024). Researchers from the London School of Hygiene & Tropical Medicine estimate more than 61,000 deaths in Khartoum State between April 2023 and June 2024 from all causes (violence, hunger, and disease)—a 50 per cent rise on the pre‐war death rate—and find evidence that the death toll across all regions is significantly higher than has been reported (Dahab et al., 2024). The United Nations (UN)'s Panel of Experts on the Sudan estimated that in 2023, in El Geneina alone, the RSF and allied militias killed between 10,000 and 15,000 people, ethnically targeting the Masalit community in particular (UNSC, 2024; see also Human Rights Watch, 2024). The weaponisation of rape and sexual violence has inflicted immense suffering on women and girls especially, with the majority of perpetrators identified as RSF or allied militia members who have used rape to terrorise communities and ethnically target non‐Arab communities in the Darfur region and Al Jazirah State, with additional violations by the SAF (Amnesty International, 2023; SIHA Network, 2025; UNHCR, 2025).

By July 2024, the fighting between the SAF and the RSF had displaced 7.9 million people within the country and a further 2.1 million had fled to neighbouring countries (UN OCHA, 2024a). The conflict has collapsed essential services in some states, with staff displaced or left unpaid, and public infrastructure has been repurposed or destroyed (Sudan Education Sector, 2023; UNICEF, 2023). Economically, the war has led to rising inflation, declining liquidity, and disruption of financial and marketing systems (ACAPS, 2023). By September 2024, after 500 days of conflict, the UN Office for the Coordination of Humanitarian Affairs (OCHA) reported that Sudan was ‘the world's largest hunger, protection, and displacement crisis’ (UN OCHA, 2024b, p. 5), with 25.6 million people suffering acute hunger, and famine conditions found in the Zamzam refugee camp in North Darfur State.

These numbers have continued to increase since we carried out our primary research. Severe access constraints mean data remain estimates only, however. The United States Special Envoy for Sudan Tom Perriello stated in May 2024 that the death toll may be as high as 150,000 (Yibeltal and Rukanga, 2024). In 2025, an estimated 64 per cent of the population, 30.4 million people, were reported to need humanitarian and protection assistance (UN OCHA, 2024c), with famine in 10 areas and 17 more at risk of it (Kinzli, 2025).^2^ In total, as of September 2025, close to an estimated 12 million people were forcibly displaced by the fighting between the SAF and the RSF, with more than four million of these people going to neighbouring countries^3^ (UNHCR, 2025). Reportedly, more than 1.3 million have started returning to their homes, mainly to Khartoum, Sennar, and Al Jazirah States (UN, 2025).

Given the nature of the crisis in Sudan and the collapse of the Sudanese state, humanitarian assistance, provided by a range of local and international actors, is now the principal form of support for those affected by the conflict or experiencing other vulnerabilities. However, the international response faces major access and capacity challenges. The UN's Humanitarian Response Plan (HRP) for Sudan was 51 per cent funded for 2023 and 70 per cent funded for 2024 (Financial Tracking Service, 2025a, 2025b). As of 7 December 2025, the 2025 HRP prioritised ask of USD 3.01 billion—a reduction from the original ask of USD 4.2 billion that left many in need unserved^4^— was only 49 per cent funded (Financial Tracking Service, 2025c).

Cross‐border assistance to Darfur from Chad began in August 2023, but for the most part during the research period the international response remained centralised in Port Sudan on the country's eastern coast. In the peripheral, historically remote regions, with their long histories of conflict, and the now‐ransacked central Khartoum, aid access and the presence of aid organisations became difficult or non‐existent.

From the start of the conflict in April 2023, agencies have been grappling with serious operational and security constraints (NRC, 2023). The warring SAF and RSF have killed, injured, detained, kidnapped, and tortured aid workers, local responders, mutual aid volunteers, and healthcare workers, especially in Al Jazirah, North Darfur, and Khartoum (ACAPS, 2025). With aid a long‐standing component of Sudan's exploitative political economy (Jaspars and Oette, 2023), both sides have captured and looted aid resources and controlled access to them, through checkpoints and blockades as well as registration requirements and fees administered by the SAF's Humanitarian Aid Commission (HAC) and the RSF's Sudanese Agency for Relief & Humanitarian Operations (the latter set up in August 2023) (ACAPS, 2023, 2025; Harvey et al., 2023b; MSF, 2023; NRC, 2023; Abdel Aziz, 2024).

When conflict erupted, the fierce fighting between the SAF and the RSF caused many Sudanese aid workers in Khartoum and elsewhere to flee, while international aid organisations laid off others when programmes were suspended (Goldberg and Ibrahim, 2023). National NGOs were hit especially hard by the warring parties and civilian gangs as order broke down: their assets were looted and staff were forced to flee (Bradbury et al., 2023; Abdel Aziz, 2024).

A prominent feature of this crisis is the relief assistance that community‐based groups have led on and delivered. Networks of mutual aid and solidarity based on familial, neighbourhood, or other social ties are critical lifelines in times of crisis in most societies, acting as informal social safety nets (Kim et al., 2022). Since April 2023, a diverse range of volunteer groups across Sudan have been providing assistance and protection in their communities, particularly those beyond the reach of external actors. Some of these initiatives are rooted in cultural regular mutual aid practices. Some evolved out of the network of resistance committees that were instrumental in securing the downfall of al‐Bashir. One well‐publicised feature of the locally‐led response during the conflict has been for assorted groups and networks to come together in ERRs in states across Sudan.

Since many of these hubs grew out of the pro‐democracy resistance committees, or work closely with them, the SAF‐ and the RSF‐controlled authorities have viewed them with suspicion, sometimes obstructing and harassing them (Nasir, Rhodes, and Kleinfeld, 2023)—attempting to control and even ban their activities, and target their leaders and volunteers—with consequent high personal risk, as described above. At the same time, the local landscape is nuanced: some ERRs and other community groups have been given financial and in‐kind support by local government authorities; some have registered with the HAC, such as in Kassala and Northern States (Abbas and Abdalhadi, 2023).

### 2.2 Debates about localisation, conflict sensitivity, and risk management

#### 2.2.1 Locally‐led aid in conflict crises

At the World Humanitarian Summit in 2016, donors and humanitarian aid organisations agreed a ‘Grand Bargain’ to make emergency aid more effective and efficient.^5^ A core commitment was ‘localisation’, empowering national and local responders by giving them more decision‐making power and resources (Grand Bargain Localisation Workstream, 2019). This agenda has persisted through successive iterations—Grand Bargain 2.0 was agreed in 2021 and 3.0 in 2023.^6^ Other initiatives building on these commitments include the Pledge for Change, launched in 2022 by Adeso and like‐minded international NGOs, with a focus on achieving equitable partnerships with national and local actors,^7^ as well as the RINGO Project,^8^ developed in 2020 by Rights CoLab and the West Africa Civil Society Institute.^9^ In practice, progress towards localisation has been limited globally (Metcalfe‐Hough, Fenton, and Manji, 2023) and with respect to Sudan (The Conflict Sensitivity Facility, 2023).

Roborgh (2023, p. 525) usefully parses the definitional complexities of the term ‘localisation’. She notes: the constructed, contested nature of ‘the local’; the unhelpfully oversimplified distinctions between ‘local’ and ‘international’; and, citing Mac Ginty (2015, p. 847), the risks of homogenising and romanticising local actors through labels that can ‘flatten out’ the realities of multiple layers, political dynamics, and ‘complex, and sometimes contradictory, identities’. We follow Baguios et al. (2021) in considering localisation as a means to an end: ‘the journey towards locally led practice’. Wall and Hedlund (2016) reserve the term ‘locally‐led’ for work that originates with local actors or is designed to support it (as distinct from that which local actors implement on behalf of others through a form of subcontracting). A decolonial critique of the aid system draws out how this journey is stymied by the racist and colonial roots underpinning the unequal power (manifested in both decision‐making and funding) between international aid organisations and local actors in the global aid system (Baguios et al., 2021).

Two particular issues from the literature frame our study: (i) the limited focus on registered organisations in localisation discourse; and (ii) the specific concerns raised by localising aid in conflict zones. As explored in Birch, Carter, and Satti (2024), on the first matter, the humanitarian localisation agenda has concentrated largely on the relationship between formally constituted bodies, such as between national and international NGOs (Kim et al., 2022). This ignores the less visible sources of assistance on which crisis‐affected populations depend, particularly those in violent conflict settings (see Harvey et al., 2023a, on Myanmar; Kim et al., 2022, on Yemen; Wall and Hedlund, 2016, on Syria). These systems of societal support operate ‘in the “background” of nearly all humanitarian responses’ (Robillard, Atim, and Maxwell, 2021, p. 34). They draw on resources from within social groups, which may be geographically extensive, particularly if the diaspora is reached (Fitzpatrick et al., 2022). Factors such as kinship, gender, location, or livelihood, together with societal power dynamics, shape informal social safety nets (Maxwell et al., 2016; Kim et al., 2020), underlining the importance of understanding risk and vulnerability in relational, not just individual, terms (Stites, Humphrey, and Krystalli, 2021). As for the second issue, localising aid in conflict raises some particular concerns. One is the concept of ‘forced’ localisation, where growing violence or political repression rather than purpose or values drive the increased responsibilities of local actors. This usually involves a rapid shift in modality (for instance, towards remote management), which does not allow for the careful reform of organisational culture and systems that ‘high‐quality’ localisation requires (Haines and Buchanan, 2023).^10^ Another concern, particularly under remote management, is that inequalities between national and international actors may widen still further as national actors are exposed to greater levels of risk in settings where compliance is harder to demonstrate, potentially reinforcing perceptions of low capacity (Barter and Sumlut, 2023).

#### 2.2.2 Conflict sensitivity

With a global rise in the number of violent and protracted conflicts, there has been increasing debate among international aid actors on how to ensure that assistance is ‘conflict‐sensitive’, that its interaction with local political and social dynamics does not inflame or incite tensions, and that, where possible, it can support peace (OECD, 2019; IASC, 2020; UNSDG, 2022). For example, a conflict‐sensitive approach to targeting responds to local perceptions of vulnerability and fairness (Humphrey, Krishnan, and Krystalli, 2019), with community‐based processes to confirm beneficiary selection and monitor exclusion (Birch et al., 2023).

A review of evidence from Kenya, Somalia, and Sudan (Birch et al., 2023) found that international social protection actors have tended to view conflict as a discrete shock, rather than as the chronic condition experienced by many countries in the Horn of Africa, including Sudan. Indeed, in this journal's 2023 issue on ‘Remembering Darfur – marking 20 years of conflict and its fallout’, a review of articles published since the 1980s on Darfur and Sudan found that recent contributions seldom explore the broader political economy or offer an in‐depth analysis of conflict (Jaspars, Abdul‐Jalil, and Pantuliano, 2023). Yet, this analysis is critical for operationalising conflict sensitivity, to comprehend the potential impacts of aid design and implementation decisions on relations between the state and society, as well as within society at various levels. Only through political economy analysis, for instance, will it be possible to understand what exclusion is driven by power‐holders and how aid interventions can mitigate this.

In the context of Sudan, our study identified the following challenges to conflict‐sensitive assistance:

1. International policy engagement has prioritised short‐term stabilisation above long‐term reform, buttressing the position of those with a vested interest in sustaining violence.
2. The parties to the conflict, the SAF and the RSF, control both aid itself and major parts of Sudan's economy, including sectors such as transportation, financial services, and telecommunications on which the aid system depends, thus increasing the risk that aid will feed conflict.
3. High levels of violence and other operational constraints are impeding not only needs assessments but also an understanding of how aid and conflict interact. As well as some areas effectively being cut off, even in some cases under siege, the retreat of international aid organisations to remote working during the crisis has widened the gap between international and local actors and the Sudanese population. This distance poses a risk to the quality of international actors' context analysis, stakeholder communication, and monitoring, all of which underpin conflict‐sensitive practice.
4. The very high levels of displacement and its diverse and fragmentary nature create particular operational challenges for agencies in terms of reaching people on the move and mitigating social tensions, which may be highly context‐specific.

#### 2.2.3 Approaches to risk

In an evolving war setting such as in Sudan, the risks involved in aid interventions will be many, serious, and fast‐changing. They may range from safeguarding issues to delivery, fiduciary, or reputational threats (Cabot Venton and Pongracz, 2021). Our research highlighted the following key risks in Sudan in late 2023 to early 2024: authorities controlling aid, including aid passing through community groups; high levels of violence and insecurity and remote working impeding understanding of conflict dynamics and needs; and external agencies co‐opting local initiatives to serve their agenda, thereby undermining their capacity to manage conflict dynamics in the way they think best. As the conflict has progressed, reports have revealed risks to the safety of aid workers and community volunteers owing to the actions of the SAF and the RSF: 25 aid workers were killed by various forces and collateral violence in 2023 (Humanitarian Outcomes, 2024), and volunteers were targeted for their frontline roles—dozens killed, others arrested (Shabaka, 2024).

Once a risk is identified, donors and other agencies will design strategies intended to diminish the possibility of the risk arising, or if it is realised, to minimise the impact. Scoones (2024, pp. 2, 112) notes how aid actors' risk management approaches range from ‘risk‐focused, techno‐managerial’ vision and methods that are control‐oriented and based on an assumption that it is possible to know about and manage the future, to approaches that are about embracing uncertainty through flexible, practice‐based approaches.

Adopting a balanced approach to risk is an important finding from the literature on complex emergencies. Yet, critical analyses find that in practice, a risk‐centred compliance approach—that is, one that focuses on financial due diligence and risk management as defined by donors in ways that detract from local capacities to respond to uncertainties and complex operational realities—has come to dominate the social protection field in more stable settings, as well as in much humanitarian assistance (Barbelet et al., 2021; Caravani et al., 2022; Robillard, Atim, and Maxwell, 2021). How donors' current preoccupation with risk is experienced by implementing partners is illustrated by the following comment by an international aid worker supporting the humanitarian response in Sudan:

*Twenty years ago, when I started work, [funding] proposals were all about logical frameworks. Today, three‐quarters of a proposal to donors is about risk management—how to deal with counterterrorism, with fraud. All this translates into bottlenecks downstream*.^11^

## 3 MATERIALS AND METHODS

This paper draws on qualitative research conducted through a literature review and key informant interviews that were mainly carried out online between June 2023 and February 2024. Our research team of three (two Northern researchers and one Sudanese researcher) focused on two research questions, as set out in the introduction: (i) what can external aid actors learn from locally‐led initiatives in Sudan to improve the conflict sensitivity of their policy and programming?; and (ii) how can official aid link with and support community mechanisms in Sudan in a conflict‐sensitive way? Our focus was on ‘social assistance’, that is, food and cash‐based transfers, which dominate the social protection portfolios of governments in low‐income countries, but which are also delivered through the humanitarian system (Sabates‐Wheeler et al., 2022; Birch et al., 2023). Our research explored a few selected examples of local and externally‐led approaches, from community‐based systems of mutual aid and solidarity to the programming of international aid agencies.^12^

We conducted a rapid review of literature published online and in English and 23 semi‐structured interviews with individuals selected from different stakeholder groups, including ERRs and other grassroots initiatives, national NGOs, Sudanese academics, international NGOs, UN agencies, and donors. Most interviews were carried out remotely, as noted, which had its limitations, particularly where the communications infrastructure was weak. This paper presents evidence from the study period (June 2023–February 2024). In the course of writing this article, we have deepened our analysis, drawing on more recent references. Although we concluded our interviews in February 2024, the issues discussed here are still relevant for the humanitarian response today in Sudan and elsewhere.

## 4 RESULTS

### 4.1 Learning from Sudanese locally‐led initiatives

Community mechanisms in Sudan have demonstrated enormous capacity to deliver social assistance, both historically and during the current crisis. They are deeply rooted in Sudanese culture and social norms and are diverse in objectives, resources, and organisation, shaped by different needs, opportunities, and capacities. Some are regular, for example the practice of *darra*, when households share food, whereas others are ad hoc, in response to drought or conflict. Traditionally village safety nets have supported poor, sick, or bereaved people, and those affected by disasters, while community assets such as classrooms and mosques are built through collective action.^13^ They enjoy a high level of public support and participation in both rural and urban settings. Urban support mechanisms have their roots in rural Sudan but have adapted in response to the needs of urban dwellers. Participation is voluntary and higher in rural areas, where social norms are stronger and where non‐participation is sanctioned by temporary exclusions (Fitzpatrick et al., 2022).

During the current crisis, this local capacity has evolved and adapted to address the new needs associated with conflict. At the time of our research (the second half of 2023 into early 2024), there were one‐off endeavours to map these efforts: Abbas and Abdalhadi (2023) mapped 28 ERRs in seven states between June and November 2023, as well as other forms of local response in six other states. Mostly unregistered, these hubs bring together entities such as resistance committees, youth and women's associations, faith groups, businesses, professional networks, and other civil society organisations. The services they provide include cash transfers, community kitchens, and food baskets. In some places, women's emergency rooms have been set up to assist pregnant and lactating women, as well as those affected by sexual violence. Here is how an ERR in eastern Sudan started and the type of services it was providing in the latter half of 2023, as relayed by a participant in our research^14^:

*We started on the first day of the war with five volunteers. The official work began on 20 April 2023 when we received one family from Khartoum. A day later, the first day of Eid, we received foreigners from different countries; some of them were students displaced from Khartoum. We were keen to make all the IDPs [internally displaced persons] feel at home. . . . We established 45 sheltering centres in total: 42 for women and children under 13 and the rest for men. Each accommodated on average 165 families. We turned the youth centre into a central kitchen and provided meals for 70 days with donations from the community. Special meals were prepared for pregnant and lactating women. . . . We also organised a campaign on social media called ‘the bid of good’ to donate milk for children. . . . Women contributed a lot to the sheltering centres in their neighbourhoods. They organised themselves into groups, with each group preparing food on a specific day. . . . The tradition of nafeer [collective work that supports individuals, households, or the public good] has been a mobilising force for human resources*.

While the members of local‐level structures we interviewed were not exposed to the term ‘conflict sensitivity’, they were not conflict‐ or peace‐blind. They endeavour to do no harm, delivering their services based on humanitarian principles and without discrimination or bias. For instance, volunteers shared information on their efforts to contact and support displaced families arriving in their area, while others indicated that they try to meet the cultural preferences, in terms of food, of different IDP groups. Moreover, they recognise the skills required for project management and are aware of the gaps in their experience, reaching out to national and international NGOs to help fill them, and some were receiving training in humanitarian principles and approaches, such as on supporting displaced people. Returning to the ERR in eastern Sudan, they relayed how they prioritise building trust within a community, including by working with embedded activists and using social media.

A critical challenge has been the overwhelming scale of the current crisis.^15^ The larger the emergency, the more likely it is that the capacity of informal social safety nets will be depleted (Fitzpatrick et al., 2022). Some ERRs have accumulated debt in their efforts to respond, while others have closed down (Abbas and Abdalhadi, 2023, p. 9). Communities in conflict zones, such as Khartoum and Darfur, have had to rely on their own resources as there is no link to local government departments and only minimal contact with aid agencies.^16^ At the time of our research, on the whole, the ERRs had limited coordination and communication within and between states, with coordinating efforts being made by the Khartoum ERRs.^17^ Many of the hubs were funded by the United States Agency for International Development in 2024; its withdrawal in 2025 is likely to have a significant impact, increasing the relevance of localised approaches.

Initiatives away from the direct violence were also affected in a number of ways. Returning again to the experience of the ERR in eastern Sudan:

*There are no military battles in our area, but the conflict has affected us in other ways. In the early weeks, the banks were shut down and mobile banking services were not working. This limited our capacity to receive donations. Communication with IDPs was also disrupted. We shared contact numbers over social media, asking IDPs to contact us, but they could not because of network issues. They would arrive and no one would be there to receive them*.

As the above description illustrates, each ERR has its particular strengths, limitations, and challenges, shaped by local political and cultural dynamics. Our research identified two key challenges for locally‐led initiatives. The first is that while governing authorities might support local initiatives with different needs, they have at times also played a disruptive role by introducing bureaucratic measures that grassroots organisations are not equipped to meet. Both the SAF and the RSF have viewed the ERRs with suspicion and have obstructed their work in places, as many grew out of the political resistance committees, or work closely with them, and therefore are seen as political rivals, committed to supporting a civilian government (Nasir, Rhodes, and Kleinfeld, 2023; Elkreem and Jaspars, 2025). For instance, after weeks of cooperation between one ERR and the local government, the government then decided to form an emergency committee that would eventually take over from the hub because the volunteers did not register it as a voluntary organisation.^18^ The aim of authorities is often to control the aid that passes through these structures, and to stop ERR activity altogether (Elkreem and Jaspars, 2025).

The second key challenge concerns the potential of local mechanisms to reinforce existing social inequalities. The most vulnerable people in society are often the least connected; their sources of support are likely to be more limited than those who enjoy more numerous and varied social connections. The participation of women and young people in these mechanisms can also be limited, particularly in areas where older men still dominate grassroots structures. Efforts by external actors to address this situation tend to produce only superficial engagement of women and young people by leaders of social safety nets in order to fulfil the requirements of external partners.^19^ Genuine change has been achieved in cases where Sudanese NGOs have used a gradual approach that recognises the structures in place and progressively starts a debate about the importance of inclusivity.^20^

### 4.2 Conflict‐sensitive support for Sudanese community mechanisms

There is increasing recognition among national and international actors of the role of informal social safety nets in responding to local shocks as well as interest in supporting the delivery of assistance through such mechanisms. At the time of our research, there were promising examples of support to ERRs from UN agencies and international NGOs, albeit in the form of small, one‐off grants. There were also positive instances of donors using fund management systems in ways that could enhance conflict sensitivity. OCHA's Sudan Humanitarian Fund (SHF) made a number of adjustments to its funding processes with the potential to reinforce conflict sensitivity, including: an area‐based approach to the reserve allocation, which means that decisions can be made by people closer to the ground; derogations that increase operational flexibility (such as applying a donation/‘no regrets’ modality to subgrants to ERRs); and allowing transfers through alternatives to the formal banking system, such as *hawalas* (informal trust‐based money transfer systems).

The representatives of Sudanese NGOs discussed how their organisations have supported informal social safety nets such as reconstruction funds since the 1990s, partially filling financial gaps and building on their capacities, structures, and experiences. During the current crisis, they have again tried to avoid creating parallel structures, and rather to work in ways that complement the activities of the ERRs.^21^

Both national and international actors noted the need to strengthen how their programming works with local NGOs and community‐based mechanisms, as a critical strategy for putting conflict sensitivity into practice, often focusing on how this is key to ‘doing no harm’. There is also awareness that supporting these local structures could have positive outcomes for inclusion and peace. Interviewees also discussed their intention to move away from transactional subcontractor relationships with national NGOs and towards more meaningful, longer‐term partnerships, sharing overheads fairly and with a lighter implementation footprint.^22^ Positive developments include the involvement of national NGOs in some international NGO decision‐making mechanisms and early stages of programme design.^23^

At the same time, some donor informants view the capacity of local actors as a constraint on localisation, in that they lack the necessary systems and qualified staff to meet the standards of accountability and due diligence which (as some acknowledge) they themselves have set, driven in part by the demands of their domestic legislatures and publics.^24^ Meanwhile, international NGOs sense that donors are reluctant to cede control and relax their requirements, such as those pertaining to the minimum documentation required.^25^ National NGOs report that some donors are more inclined to listen than others, but also that an undifferentiated view of Sudanese civil society prevails, which feeds a lack of trust in their competence and experience.^26^

Supporting Sudanese community mechanisms in a conflict‐sensitive manner requires understanding, and responding to, the risks faced by these local initiatives and their volunteers. In particular, this necessitates consideration of how international support can minimise, and not worsen, the risk of predatory and violent reactions by the Sudanese controlling authorities, both the SAF and the RSF. One potential flashpoint is registration, which has been used by both sides as a way to exercise control over the community groups, as often seen in other crisis‐affected contexts. Initiatives that need to maintain a low profile, or have no wish to legitimise a regime, will avoid registering; in some settings, however, it has been common for donors and intermediaries to require registration of their national partners, despite the risks involved for those local actors, such as in Myanmar (Haines and Buchanan, 2023). In Sudan, some international aid actors have resolved this matter by providing cash transfers to community groups which do not require them to be registered (a modality termed ‘group cash transfers’). Moreover, with the warring parties harassing and even killing community volunteers for their aid activities owing to suspicions of political allegiances and ambitions, some international organisations have provided security and protection training, albeit this has been limited and uncoordinated—for more on this and other points, see Shabaka (2024) and Carter and Satti (2025).

## 5 DISCUSSION

### 5.1 The relationship between localisation and conflict sensitivity

Our research has drawn out the complex relationship between localisation and conflict sensitivity. First, localisation—understood as progress towards locally‐designed and ‐managed aid, as discussed earlier—may either strengthen or undermine the conflict sensitivity of assistance depending largely on how it is approached and designed (see Table 1).

**TABLE 1:** Localisation and conflict sensitivity.

Circumstances under which localisation may strengthen conflict‐sensitive assistance	Circumstances under which localisation may undermine conflict‐sensitive assistance
Where the particular strength of local actors in responding holistically within their community and thus working across the humanitarian–development–peacebuilding nexus is recognised and valued. Where there are deliberate efforts to involve a diversity of actors that may not ‘look like’ NGOs but may have greater reach into remote and volatile areas and be well placed to strengthen social cohesion. Where local actors strive for inclusive, accountable assistance that is built on relationships of trust with communities and considers their perspectives. Where donors are willing to review their normative frameworks to give local actors as much flexibility and autonomy as possible. Where funding processes support core capacities and long‐term partnerships between local actors, aid agencies, and donors.	Where power and decision‐making are retained by distant actors that may be less well placed to analyse and assess conflict risks. Where the analysis and consultation that inform programming are cursory and the quality of monitoring and intelligence is poor. Where external partners consider only their relationship with a particular organisation and not the impact of their decisions on the broader civil society ecosystem. Where local actors are forced to adapt in ways that weaken what makes them effective in a conflict zone, such as their contextual knowledge, networks, and holistic adaptive practice.

**Source:** Birch, Carter, and Satti (2024, p. 22).

Second, the bedrock of conflict sensitivity is a thorough understanding of conflict and power dynamics—that is, comprehension of localised power (im)balances and shifts in control, and of the implications for who is empowered and who is marginalised in a community or an area—and how an intervention is likely to interact with them. This type of conflict‐sensitive approach can in turn inform the localisation of aid by guiding things such as partner assessment and selection. For example, what is the positionality of a NGO selected to receive international support in the power dynamics of a particular community or region, and how will this be affected when the organisation receives that support? National and international aid actors underlined in our interviews the need to inform aid design through analysis of local leaders' interests and engagement with aid, as well as national political and conflict dynamics.

A key component of conflict‐sensitive localisation in Sudan is the important ‘bridging’ role played by national NGOs between the formal aid system and grassroots structures. During the conflict, our research has drawn out how this role has been affected by several constraints. Remote working during the crisis has widened the gap between international and local actors and the Sudanese population. National NGOs have lacked a financial ‘cushion’ to manage the impacts of conflict in ways that are easier for international NGOs, including one to cover the relocation of staff or to make duty of care payments and replace lost equipment. International aid actors have also lacked trust in national NGOs, with complicated grant application processes impeding their access to support. This is underpinned by the longer‐term failure to invest adequately in the organisational capacities of national NGOs.^27^ The principal frustration of our two national NGO informants was that while they understand the context far better than international NGOs and donors, they remain relegated to a subsidiary position in the aid system. Since the basis of conflict sensitivity is an accurate analysis of conflict dynamics, they believe that local actors should guide debates on the subject in Sudan to ensure appropriate contextualisation.^28^

Other critical challenges for the localisation and conflict sensitivity of externally supported aid in Sudan were highlighted by international aid agency representatives. First, the shortage of funds in the face of large‐scale need makes it hard to select who should receive support, heightening the risk of fuelling tensions between those who benefit and those who do not.^29^ Second, aid agencies grapple continuously with staff shortages and capacity limitations (WFP, 2022), impeded by short‐term donor funding that feeds a constant cycle of recruitment.^30^ Third, the pressures of a large‐scale, fast‐paced, and logistics‐led operation make it an achievement even to deliver a minimalist approach to analysis and the mitigation of risk.^31^ Relatedly, thinking and working politically is still unfamiliar and uncomfortable terrain for some humanitarians.^32^ There are concerns that conflict sensitivity may compromise humanitarian principles or delay a response. In fact, ‘politically informed impartiality’ (Stephen et al., 2017, p. 42) is vital in situations where both national and international actors may politicise humanitarian assistance.

### 5.2 A balanced approach to managing risk

In complex crises such as the one in Sudan, where access is constrained and decision‐makers are removed from operational realities, a donor's instinct may be to manage the risks and uncertainties inherent in their support by tightening systems and controls. Yet, an overly restrictive approach to risk can widen the gap between aid agencies and the populations they serve (Weigand and Andersson, 2019) or squeeze out smaller organisations that look less like Western NGOs but might be ‘more in tune with community needs’ (Stephen et al., 2017, p. 5). External agencies may compromise the independence of local initiatives by co‐opting them to serve their agenda, and in so doing undermine their capacity to manage conflict dynamics in the way they think best. Both the literature and our interviews suggest that the opposite is required: a willingness to trust frontline aid actors and to give them as much autonomy as possible to adjust their work as the context changes, within an approach that seeks to understand the risks they face, including from external engagement, and supports their management and mitigation of those dangers. Such an approach will, however, require international aid actors to rectify their failure to address the fundamental change needed to localise aid: to transfer power to local actors (Baguios et al., 2021).

An important step towards ensuring a balanced approach to managing risk will be to review assumptions regarding the capacity of local actors. Similar to global research (Mac Ginty, 2015; Barbelet et al., 2021), we argue that it may be international actors' limited understanding of local capacity and where it exists that is an impediment to trusting and supporting locally‐led aid. Our research revealed that comprehension of locally‐specific capacities, as well as risks and how they are managed by locally‐led responses, in addition to the holistic needs of communities, can be facilitated by bringing coordination and decision‐making structures closer to crisis‐affected populations, as introduced by the SHF.

One area of risk that demands particular attention in Sudan is the interaction between official aid and the war economy; in particular, ensuring that agencies understand who they are working with and the links these companies or individuals may have with security or political actors (Hoffmann, 2022; Jaspars and Elkreem, 2023). Managing this risk need not impose significant demands on any one organisation since information about company profiles and connections can be pooled and is already being gathered (Cartier, Kahan, and Zukin, 2022).^33^

## 6 CONCLUSION

Grassroots organisations in Sudan have proved their worth in appalling circumstances. The trust they enjoy at the local level, as well as their flexibility and understanding of conflict dynamics, qualify them to play a leading role in programming. Grasping the opportunity to help them do so will not only augment the response to the dire humanitarian situation but will also reinforce locally‐rooted models of social assistance. ERR members interviewed for this study were open to such partnerships, and in some cases were already actively seeking or benefiting from them; however, they emphasised the importance of mutual trust, transparency, and accountability to making them work. Meanwhile, as discussed above, the impact of the war in terms of increasing international aid actors' remoteness and how they respond to risks in the pressured operational environment has led to shifting power dynamics between the local and the global to the detriment of local actors' share of decision‐making.

Partnerships between the official aid system and locally‐led mutual aid mechanisms will require:

1. **Analysis:** to understand the Sudanese political (war) economy, the central role of the battle for resources, and the interactions with aid.
2. **Investment in intermediaries:** to empower trusted national NGOs, which act as a bridge between the formal aid system and grassroots structures, and have the long‐term presence and capacity to strengthen grassroots leadership over time.
3. **Decentralised aid coordination and decision‐making:** to bring this closer to crisis‐affected populations and enable recognition of locally‐specific conflict risks and the holistic needs of communities.

In sum, as we have shown in this paper, there are multiple linkages between achieving localisation commitments and approaches to conflict sensitivity and risk management. Many recommendations on operationalising conflict‐sensitive assistance intersect with those needed to strengthen the role of local actors in the aid system. Meanwhile, a conflict‐sensitive approach to localisation will deepen contextual understanding and reinforce the capacities of Sudanese actors best placed to mitigate the impact of conflict over the longer term. Achieving this requires international aid actors to reassess their approach to risk. This should include considering what harms may be incurred by continuing to centralise control over interventions, as well as what opportunities to strengthen local capacities may be lost in doing so.

## CONFLICT OF INTEREST STATEMENT

The authors have no conflicts of interest to declare.

## ETHICS STATEMENT

The research was approved by the Institute of Development Studies' research ethics process.

## FUNDING STATEMENT

The research study was funded by Irish Aid and the writing of this journal article was funded by the United Kingdom's Foreign, Commonwealth & Development Office under the Better Assistance in Crises (BASIC) Research programme.

## Data Availability

Research data are confidential.

## Endnotes

^1^
Funded by Irish Aid, this is one study in a series of research projects on conflict‐sensitive social protection in the Horn of Africa and Sudan. The full research findings from the Horn of Africa and Sudan studies are published in two Institute of Development Studies Working Papers (Birch et al., 2023; Birch, Carter, and Satti, 2024). In this paper, we present a synthesis of selected findings from the Sudan case study (Birch, Carter and Satti, 2024) while bringing to bear some further critical reflections based on a wider reading of relevant literature.

^2^
Analysis published in December 2024 by the Integrated Food Security Phase Classification (IPC) ‘projected that 24.6 million people would face high levels of acute food insecurity (IPC Phase 3 or above) through May 2025, including at least 638,000 people in IPC Phase 5 (Catastrophe) and 8.1 million people in IPC Phase 4 (Emergency). The Famine Review Committee (FRC) detected Famine in five camps/areas in El Fasher and the Western Nuba Mountains, and projected that Famine would expand to five additional localities of North Darfur by May 2025. A risk of Famine was detected in 17 other locations in Central Nuba Mountains, South, East, and North Darfur, Al Jazirah, and Khartoum states’ (IPC, 2025, p. 1).

^3^
As of September 2025, data recorded since the outbreak of the violence reported 7.5 million internally displaced persons and close to 271,000 relocated refugees in Sudan and 3.3 million Sudanese refugees and slightly more than 833,000 returned refugees had crossed into neighbouring countries (UNHCR, 2025).

^4^
In May 2025, the reprioritised HRP reduced the ask from USD 4.2 billion to USD 3.01 billion, decreasing the number of people it sought to help from 20.9 million to 18 million (out of the estimated 30.4 million people in need) (UN OCHA, 2025).

^5^
For more information, see https://interagencystandingcommittee.org/grand-bargain (last accessed on 27 November 2025).

^6^
For more information, see https://www.calpnetwork.org/cash-and-voucher-assistance/policy-and-funding/grand-bargain/ (last accessed on 27 November 2025).

^7^
For more information, see https://pledgeforchange2030.org/pledges/equitable-partnerships/ (last accessed on 27 November 2025).

^8^
Re‐Imagining the INGO (International NGO) and the Role of Global Civil Society.

^9^
For more information, see https://wacsi.org/from‐innovation‐to‐transformation‐how‐ringo‐is‐revolutionising‐global‐civil‐society/#:~:text=RINGO%20is%20a%20leading%20globally%20coordinated%20cross%2Dsectoral,purpose%2C%20structures%2C%20power%2C%20and%20positioning%20of%20INGOs.&text=RINGO%20will%20therefore%20position%20WACSI%20at%20the,at%20improving%20the%20global%20civil%20society%20ecosystem (last accessed on 27 November 2025).

^10^
‘High‐quality’ localisation is motivated by solidarity (Barter and Sumlut, 2023) and concerned with ‘ethical partnership’ (Haines and Buchanan, 2023), particularly the distribution of power between partners (Stephen et al., 2017). ‘Low‐quality’ localisation is essentially subcontracting (Barter and Sumlut, 2023).

^11^
Interview with an aid worker, 29 August 2023.

^12^
For details of which interventions are included in our review, see Birch, Carter, and Satti (2024).

^13^
Interview with a member of a Services and Change Committee in eastern Sudan, 9 July 2023. These committees replaced the Popular Committees after the 2019 revolution, but they have not been constitutionally endorsed.

^14^
This is an abridged version; the complete write‐up is available in Birch, Carter, and Satti (2024). The experience of this one ERR was included in our research as an exemplary case which resonates with experiences elsewhere. Abbas and Abdalhadi (2023) provide a similar account from the Kosti ERR in White Nile State.

^15^
Interview with a member of an ERR in eastern Sudan, 23 August 2023.

^16^
Interview with a member of a community‐funded feeding programme in Khartoum, 5 August 2023.

^17^
For more information, see https://khartoumerr.org/ (last accessed on 28 November 2025).

^18^
Interview with a member of an ERR in eastern Sudan, 23 August 2023.

^19^
Interview with a Sudanese academic, 8 August 2023.

^20^
Interview with the executive director of a national NGO, 10 August 2023.

^21^
Two interviews with senior staff of Sudanese NGOs, 10 August 2023.

^22^
Interviews with a UN expert, 25 July 2023, an aid worker, 29 August 2023, and a country director, 5 September 2023.

^23^
Interview with the executive director of a national NGO, 10 August 2023.

^24^
Interviews with a donor representative, 31 January 2024, and a government humanitarian representative, 5 February 2024.

^25^
Interviews with an aid worker, 29 August 2023, and a country director, 5 September 2023.

^26^
Two interviews with senior staff of Sudanese NGOs, 10 August 2023.

^27^
Interviews with a UN expert, 26 July 2023, the director of a national NGO, 10 August 2023, and a UN agency country staff member, 19 August 2023.

^28^
Two interviews with senior staff of Sudanese NGOs, 10 August 2023.

^29^
Interviews with a UN expert, 26 July 2023, and the executive director of a national NGO, 10 August 2023.

^30^
Interview with a UN agency country staff member, 19 August 2023.

^31^
Interviews with a UN expert, 25 July 2023, and a UN agency country staff member, 19 August 2023.

^32^
Interview with a UN expert, 25 July 2023.

^33^
For more information, see https://c4ads.org/news/sudan-conflict-response/ (last accessed on 28 November 2025).
